# Resveratrol given intraperitoneally does not inhibit the growth of high-risk t(4;11) acute lymphoblastic leukemia cells in a NOD/SCID mouse model

**DOI:** 10.3892/ijo.2011.1316

**Published:** 2011-12-22

**Authors:** SUSAN J. ZUNINO, DAVID H. STORMS, JOHN W. NEWMAN, THERESA L. PEDERSEN, CARL L. KEEN, JONATHAN M. DUCORE

**Affiliations:** 1 United States Department of Agriculture, Agricultural Research Service, Western Human Nutrition Research Center, 430 West Health Sciences Drive; 2 Department of Nutrition, University of California Davis, One Shields Ave., Davis, CA 95616; 3 Department of Pediatrics, Section of Hematology/Oncology, University of California School of Medicine, 2516 Stockton Blvd., Sacramento, CA 95817, USA

**Keywords:** t(4;11) acute lymphoblastic leukemia, resveratrol, vincristine, metabolites, NOD/SCID mice

## Abstract

The efficacy of resveratrol as a preventive agent against the growth of t(4;11) acute lymphoblastic leukemia (ALL) was evaluated in NOD.CB17-Prkdcscid/J mice engrafted with the human t(4;11) ALL SEM cell line. SEM cells were injected into the tail vein and engraftment was monitored by flow cytometry. Once engraftment was observed, mice were injected intraperitoneally with resveratrol (10 mg/kg body weight) dissolved in dimethylsulfoxide (DMSO) or DMSO alone (control) every other day, or vincristine (0.5 mg/kg body weight) 3 times per week for 4 weeks (n=16 per group). Comparisons of the percent of human leukemia cells in blood and survival curves showed resveratrol did not inhibit progression of the disease. Liquid chromatography-tandem mass spectrometry analyses of mouse sera showed resveratrol was rapidly metabolized to glucuronidated and sulfated forms 1 h post-injection, with low to no resveratrol or metabolites observed in sera by 24–48 h. These data indicate that in contrast to findings in *in vitro* models, parenterally administered resveratrol does not have potential as a preventive agent against high risk t(4;11) ALL.

## Introduction

Resveratrol (3,5,4′-trihydroxy-*trans*-stilbene) is a member of the phytoalexin class of antibiotics and its production can be induced in a variety of plants in response to fungal infections, environmental stress, or injury (1). Resveratrol is a well-known component of the skin of grapes, is present in high concentrations in red wine, and is also found in other plant foods including blueberries, mulberries, rhubarb, and cranberries. Jang *et al* (2) were the first to describe the ability of resveratrol to inhibit events associated with the initiation, promotion, and progression of cancer. Subsequent to the report by Jang *et al*, numerous *in vitro* and *in vivo* investigations have provided support for the concept that resveratrol may be efficacious in the prevention of certain cancers. Resveratrol has been shown to act as a chemopreventive agent against chemically-induced mammary and esophageal carcinogenesis in rats (3,4), lung cancer and transplanted liver tumors in mice (5,6), and has been reported to suppress the growth of aberrant crypt foci in the colon (7). Resveratrol given to A/J mice at a daily intraperitoneal (i.p.) dose of 40 mg/kg body weight for 28 days significantly inhibited the growth of subcutaneously xenografted neuroblastoma cells in mice (8). A 6-fold decrease in tumor volume and a significantly improved survival rate (70%) were observed in the mice receiving resveratrol compared to control mice. Resveratrol has been shown to inhibit proliferation and induce apoptotic cell death in a number of different types of cancer cells *in vitro*. Numerous reports have been published on the potential mechanisms that might contribute to the anti-cancer activity of this compound (1,9–11). Mechanistic studies have revealed that resveratrol can act as an antioxidant, inhibit transcription factor activation, and inhibit kinase pathways involved in cell signaling, including those involved in progression of the cell cycle.

Acute lymphoblastic leukemia (ALL) with chromosomal translocation t(4;11) is a highly aggressive leukemia found in 60–85% of infants, 2% of children, and 3–6% of adults with ALL (12,13). The presence of this translocation is strongly associated with poor responses to conventional chemotherapeutic agents, relapse, and a poor prognosis for survival. The mechanisms underlying the malignancy of t(4;11) ALL are poorly understood. Defective or dysregulated apoptotic pathways during hematopoiesis may play a role in the generation of these leukemias. Several cell lines have been established from patients with ALL carrying the t(4;11) (q21;q23) chromosomal translocation, and these lines provide useful tools to evaluate the efficacy of alternative preventive and therapeutic strategies (14–16).

The nonobese diabetic/severe combined immunodeficient (NOD/SCID) mouse model has been successfully used to examine chemotherapeutic strategies against hematopoietic cancers (17–20). It was shown that engraftment of ALL in these mice mimics the human disease by homing to bone marrow, spleen, and liver, with significant presentation in peripheral blood (18,21). We have shown that resveratrol can effectively induce apoptotic cell death *in vitro* in cell lines that were established from patients with ALL that carry the t(4;11) (q21;q23) chromosomal translocation, as well as other ALL lines without the translocation (22). We hypothesized that resveratrol would be efficacious in the treatment of high-risk t(4;11) ALL *in vivo*. In the present study, we evaluated the efficacy of parenterally administered resveratrol in the NOD/SCID mouse model after engrafting the SEM cell line that carries the t(4;11) translocation. Resveratrol was compared to a standard chemotherapeutic agent, vincristine, that is used in clinical settings to treat t(4;11) ALL (23). Serum levels of resveratrol and metabolites were measured by liquid chromatography/mass spectrometry (LC/MS) to determine whether parenteral administration would provide the levels of resveratrol needed to prevent the growth of this leukemia.

## Materials and methods

### Cells and reagents

SEM is an established cell line from a patient diagnosed with high-risk pre-B ALL containing the chromosomal translocation t(4;11)(q21;q23) (14). The cells were grown at 37°C, 5% CO_2_ in RPMI-1640 (Invitrogen, Carlsbad, CA) supplemented with 10% fetal bovine serum (Sigma, St. Louis, MO), 50 IU/ml penicillin, 50 μg/ml streptomycin, 0.25 μg/ml amphotericin B, 1 mM sodium pyruvate, and 2 mM L-glutamine (Invitrogen). For injection into mice, SEM cells were harvested, washed twice in Dulbecco’s phosphate-buffered saline (PBS) without Ca^2+^ or Mg^+^ (Sigma), and resuspended at a final concentration of 50×10^6^ cells/ml in PBS.

Vincristine sulfate and resveratrol (>99% pure) were purchased from Sigma. Vincristine sulfate was dissolved in PBS. Resveratrol was dissolved in dimethylsulfoxide (DMSO, Sigma). The solutions were filter sterilized, aliquoted, and frozen at −20°C until use. Phycoerythrin-cyanin 7 (PE-Cy7)-conjugated anti-human CD19, allophycocyanin-Cy7 (APC-Cy7) conjugated anti-mouse CD45 were purchased from Becton-Dickinson (San Jose, CA). The trans-isomers of resveratrol, tetra-deuterated resveratrol (d4-resveratrol), resveratrol-3-O-D-glucuronide, resveratrol-4′-O-D-glucuronide, and resveratrol-3-O-sulfate, and 1-cyclohexyluriedo-3-dodecanoic acid (CUDA) were obtained from Cayman Chemical Co. (Ann Arbor, MI). Sulfatase from *Aerobacter aerogenes*, β-glucuronidase (Type IX-A) from *Escherichia coli*, formic acid, glycerol, potassium 4-nitrophenyl sulfate, and 4-nitrophenyl β-D-glucuronide were purchased from Sigma. Ammonium hydroxide and LC/MS grades of methanol, acetonitrile, and water were obtained from Fisher Scientific (Fair Lawn, NJ). Normal mouse serum was obtained from United States Biological (Swampscott, MA).

### Immunodeficient NOD/SCID mice

All experimental procedures were approved by the University of California Davis Institutional Animal Care and Use Committee. Five- to six-weeks-old female NOD.CB17-Prkdc^scid^/J mice were purchased from the Jackson Laboratory (Bar Harbor, ME, common name NOD/SCID). Mice were housed and handled under pathogen-free conditions at the University of California, Davis vivarium in a temperature controlled environment with a 12-h light-dark cycle. Mice were fed a commercial rodent diet (Diet 7013, Harlan Teklad, Madison, WI) that was sterilized by gamma irradiation. Mice were given sterilized food and water *ad libitum*. Mice were weighed once per week in a biosafety cabinet to maintain pathogen-free conditions. At the age of 8 weeks, each mouse was injected with 5×10^6^ SEM cells through the tail vein using a 1-cm^3^ syringe with a 30-G needle (Becton-Dickinson). The injection volume was 100 μl.

### Detection of leukemia cell engraftment

Beginning 2 weeks after the tail vein injections of leukemia cells, ~50 μl of blood was collected from the tail artery of each mouse once per week to monitor engraftment of the human leukemia cells. Blood was collected directly into heparinized Microvette tubes (Sarstedt, Newton, NC) and transferred to 1.5 ml microfuge tubes, where red blood cells were lysed using PharmLyse (Becton-Dickinson) according to the manufacturer’s recommendation. The peripheral blood leukocytes (PBLs) were stained with PE-Cy7 conjugated anti-human CD19 and APC-Cy7 conjugated anti-mouse CD45 at room temperature for 20 min. The cells were washed in PBS containing 0.1% BSA and 7 mm sodium azide (Sigma) and then fixed in 1% paraformaldehyde (Sigma) before analysis by flow cytometry. The stained cells were analyzed on a FACSCanto™ fluorescence-activated cell sorter (FACS) using FACSDiva™ software (Becton-Dickinson). Each analysis of peripheral blood cells was performed using appropriate scatter gates to exclude cellular debris and aggregated cells. PBLs prepared from NOD/SCID mice not injected with leukemia cells were used as a negative control for engraftment. These cells were frozen at −80°C in 10% DMSO, 90% fetal bovine serum until use. As a positive control for CD19^+^ cells, SEM cells were added to an aliquot of thawed PBLs from non-engrafted mice. The negative and positive control cells were stained as described above and used to set the gates for human CD19^+^ cells. Thirty thousand events were collected for each sample. Positive engraftment was established when the proportion of human CD19^+^ cells reached 1% in the murine PBL population (17,18).

### Initial treatment with resveratrol and toxicity assessment

Once engraftment of leukemia was observed in the peripheral blood, mice were randomly separated into control, resveratrol, and vincristine treatment groups (n=13–14 per group). The mice were treated daily with i.p. injections of DMSO and resveratrol (40 mg/kg body weight), or once per week with vincristine (0.5 mg/kg body weight). The 40 mg/kg dose of resveratrol was chosen because it was reported to be effective against neuroblastoma in mice (8). The approximate volume per injection was between 80–120 μl depending upon the weight of the mouse. During the treatments, the blood from each mouse was monitored for growth of the leukemia cells by flow cytometry. Body weights were obtained weekly in order to adjust the quantity of chemical per animal.

The 40-mg/kg dose of resveratrol proved toxic to the leukemic NOD/SCID mice, requiring a toxicity analysis. Sixteen mice were separated into groups of 4 mice each and treated i.p. daily with DMSO, or doses of resveratrol at 5, 10, or 20 mg/kg body weight for one week. After one week, the mice were injected with the same doses once every other day for 3 weeks. The mice were monitored daily for signs of illness.

### Treatment with resveratrol at a lower concentration

Forty-eight mice (age of 8 weeks) were injected with SEM leukemia cells as described above. Once engraftment of leukemia was observed in the peripheral blood by flow cytometry, mice were randomly separated into control, resveratrol, or vincristine treatment groups (n=16 per group). The mice were treated every other day with i.p. injections of DMSO and resveratrol (10 mg/kg body weight), or three times per week with vincristine (0.5 mg/kg body weight). The mice were treated for 4 weeks. Body weights and percent of human CD19^+^ cells in the mouse PBMC population were measured weekly. For this experiment, volumes of DMSO and resveratrol were reduced to 40–60 μl per mouse according to body weight. Injection volumes of vincristine were between 80–130 μl per mouse.

### Analysis of engraftment sites

Blood, spleens, and bone marrow were harvested from 4 mice from the DMSO treatment group following euthanasia to confirm engraftment sites of the SEM leukemia cells. PBLs were prepared as described above. Spleens were removed, placed in RPMI medium, and perfused with medium using a syringe and 25 G needle. The spleens were then shredded using the end of the needle, the cells that were released into the medium were collected, and the red blood cells were lysed with PharmLyse. Both femurs from each mouse were placed into RPMI medium and the ends of the femurs were cut. Bone marrow was removed by perfusing the inside of the bone with medium using a 27-G needle with syringe. PBLs, splenocytes, and bone marrow cells were stained with PE-Cy7 conjugated anti-human CD19 and APC-Cy7 conjugated anti-mouse CD45 and analyzed by flow cytometry as described above.

### Quantification of resveratrol and resveratrol metabolites

Protocols optimizing resveratrol metabolite deconjugation and resveratrol extraction from mouse sera, and ultra performance liquid chromatography-tandem mass spectrometry (UPLC-MS/MS) quantification were developed for this study. Serum from each mouse was removed from −70°C, thawed on ice, and separated into three 25-μl aliquots. Each aliquot was spiked with nitrophenyl glucuronide and nitrophenyl sulfate, each at a final concentration of 0.5 μM as digestion controls, and d4-resveratrol at a final concentration of 2 μM as a recovery surrogate. Positive controls were prepared in each analytical batch from three 25 μl aliquots of commercially available normal mouse serum prepared as above, which also received resveratrol, resveratrol-3-sulfate, and the two resveratrol glucuronides at the final concentrations shown in Table I. The aliquots from each triplicate set were then spiked with either 5 μl 0.1 mM ammonium formate pH 6.9 (mock digest), 5 μl (0.5 kU) β-glucuronidase reconstituted in formate buffer, or 10 μl sulfatase (0.11 U) as supplied. Samples were incubated at 37°C for 1 h in an orbital water bath (Boekel, Feasterville, PA) at 60 Hz protected from light. Reactions were chilled and quenched with 100 μl cold acetonitrile using a 5 min 4°C vortex. Samples were further chilled at −20°C for 10 min to assist protein precipitation, and centrifuged at 14,000 × g for 10 min at 4°C. Sample supernatants were removed to a screw capped polypropylene tube containing 5 μl 50% methanolic glycerol, pellets were re-extracted with 100 μl acetonitrile, and extracts were pooled. The extracts were dried using a Savant SV110A SpeedVac (Savant Instrument Inc., Holbrook, NY) and reconstituted in 100 μl 100 nM CUDA in methanol. After vortexing, the reconstituted extracts were filtered with 0.1 μm Amicon Ultrafree-MC durapore PVDF filter (Millipore, Billerica, MA) for 4 min at 4,000 × g and transferred to a glass insert in 2 ml amber vials, capped, and stored at 4°C for LC-MS/MS analysis. In addition, the digestion and extraction controls described above, each extraction batch also contained mock digested, unspiked aliquots of normal mouse serum samples whose extracts were enriched with target analytes just before filtration. These samples served as a ‘matrix normalization solution’ allowing assessment of matrix effects upon the analyte detection. Methanol normalization solutions were prepared by spiking 100 μl of 100 nM CUDA with 1 μl of the spike used for matrix normalization solutions, and were analyzed as a further control. With each batch of samples, a seven point standard curve was constructed ranging from 1 to 3,000 nM of resveratrol, resveratrol sulfate and glucuronides, and nitrophenyl sulfate and glucuronide in a methanolic solution of 100 nM CUDA and 2 μM d4-resveratrol.

Chromatographic separation was achieved using a gradient of acidified water and acetonitrile on a 2.1×150 mm, 1.7 μm Acquity UPLC BEH C_18_ column on an Acquity ultra performance liquid chromatograph (UPLC, Waters Corp., Milford, MA). Sample were held at 10°C and aliquots (10 μl) were injected on to the 50°C column equilibrated with 90/10 v/v 0.1% formic acid:acetonitrile. Initial conditions were held for 1 min, ramped to 65% acetonitrile at 7 min, 95% acetonitrile at 8 min and held for 2 min. Solvent flow was 0.25 ml/min. Mass spectral analysis of the UPLC effluent was performed with an API 4000 QTrap tandem mass spectrometer (AB SCIEX, Foster City, CA) using negative electrospray ionization (ESI-) in multiresidue mode (MRM). Optimized analyte parameters are summarized in Table I. Data analysis was performed using Analyst 1.4.2 (AB SCIEX). All analyte signals were measured as peak area ratios to the internal standard, CUDA, to correct for variability in sample volume, injection volume, and instrumental drift. Resveratrol serum concentrations were determined from 1/x weighted linear regressions of methanolic standard curves described above. Reported serum resveratrol concentrations are corrected for procedural losses by dividing CUDA-linked results by the fractional recovery of d4-resveratrol in each sample. The limit of detection of resveratrol was based on visually defined peaks with a signal to noise ratio >2 (24).

### Statistical analysis

For statistical comparisons between treatment groups, the event-free survival (EFS) was calculated beginning with the initiation of treatment. An event was defined as overt clinical illness necessitating euthanasia, which included >20% weight loss, lethargy, severe weakness, or inability to reach food or water for 24 h. All statistical analyses were performed with GraphPad software (GraphPad Software, Inc., San Diego, CA) and data are displayed as arithmetic means ± standard deviation (SD), unless otherwise noted. One-way and two-way analysis of variance (ANOVA) were used to compare body weights, percent engraftment at the beginning of treatment, and percent CD19^+^ cells over time with Bonferroni post-tests. EFS was analyzed by Kaplan-Meier plots with differences calculated using the log-rank test. Differences were considered significant for α (p<0.05).

## Results

### Resveratrol toxicity in leukemic mice

**Figure 1:**
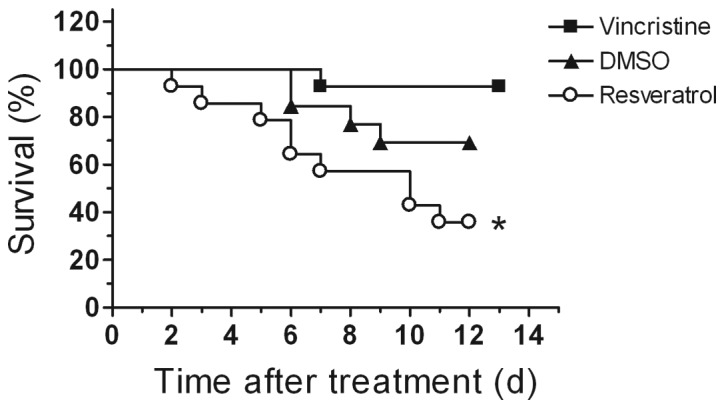
Resveratrol at a concentration of 40 mg/kg body weight was toxic to NOD/SCID mice engrafted with t(4;11) acute lymphoblastic leukemia (ALL). Mice were engrafted with the t(4;11) ALL line SEM, and then treated intraperitoneally with either DMSO (n=13) or resveratrol (40 mg/kg body weight, n=14) daily, or vincristine (0.5 mg/kg body weight, n=13) one time per week for 4 weeks. Mice were euthanized when they became clinically ill, i.e., showed >20% weight loss, lethargy, weakness, or inability to reach food or water. Differences in survival after treatment began were determined by log-rank test. The asterisk indicates a significant reduction in survival for resveratrol-treated mice compared to the DMSO and vincristine treated mice (p<0.05).

Engraftment in the mice as determined by flow cytometry was 100% for both of the engraftment/treatment experiments. A 40-mg/kg body weight dose of resveratrol was used successfully to treat subcutaneously xenografted neuroblastoma cells in mice (8). Therefore, we used this dose in our initial experiments. However, with daily i.p. injections of 40 mg/kg resveratrol, survival of the leukemic mice was greatly reduced compared to both the DMSO control and vincristine-treated mice (Fig. 1, p<0.05, log-rank test). Approximately 36% of mice in the resveratrol group survived compared to 69% in the DMSO control group and 93% in the vincristine group by day 12 of treatment. The experiment was ended on day 13 of treatment and all mice were euthanized.

A toxicity test with lower doses of resveratrol was performed to determine the highest acceptable dose for treatment. Doses of resveratrol at 5, 10, and 20 mg/kg body weight were evaluated in non-leukemic NOD/SCID mice. Mice were injected i.p. daily for one week. We found that these mice were intolerant to the 80–120-μl volumes of DMSO, and exhibited temporary weakness in the hindlimbs after injections. Therefore, after the first week of daily injections, the treatment was changed to every other day and was continued for a further 3 weeks. A resveratrol dose of 10 mg/kg body weight in a 40–60-μl volume of DMSO administered every other day was determined to be the optimal volume and concentration that did not show toxicity (data not shown).

### Intraperitoneal administration of resveratrol does not kill t(4;11) ALL

**Figure 2:**
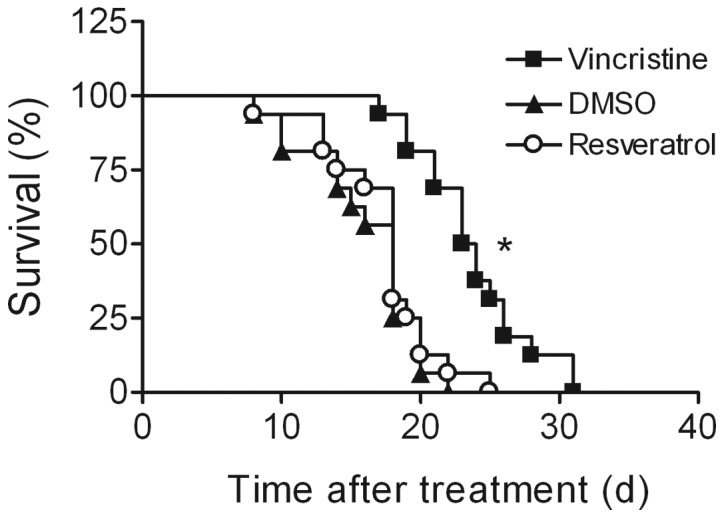
Resveratrol at a concentration of 10 mg/kg body weight did not increase survival of leukemic mice. Three groups of mice were engrafted with the t(4;11) ALL line SEM, and then treated intraperitoneally with either DMSO or resveratrol (10 mg/kg body weight) every other day, or vincristine (0.5 mg/kg body weight) three times per week for 4 weeks (n=16 per treatment group). Mice were euthanized when they became clinically ill as described in Fig. 1. Differences in survival after treatment began were determined by log-rank test. The asterisk indicates a significant difference in survival between vincristine and DMSO or resveratrol treated mice (p<0.05).

Resveratrol was administered i.p. at a dose of 10 mg/kg body weight to leukemic mice every other day. To reduce the hindlimb weakness that was observed with the DMSO, the injection volumes of the DMSO control vehicle and resveratrol were reduced by half (injection volumes between 40–60 μl) for this study. These smaller volumes reduced signs of discomfort and weakness in the mice. The mean percents of human CD19^+^ cells in the mouse PBL population at the beginning of treatment were not different between the groups of mice (DMSO control group was 3.5±3.0%; vincristine group was 1.9±1.1%; resveratrol group was 2.5±2.1%, p>0.05). Treatments began between 3–4 weeks after the injection of the leukemia cells into the mice (age 11–12 weeks). After 4 weeks of treatment, survival curves show that resveratrol was similar in efficacy to the DMSO control (Fig. 2). Treatment with vincristine increased survival of the mice compared to the control mice (p<0.05, log-rank test).

**Figure 3:**
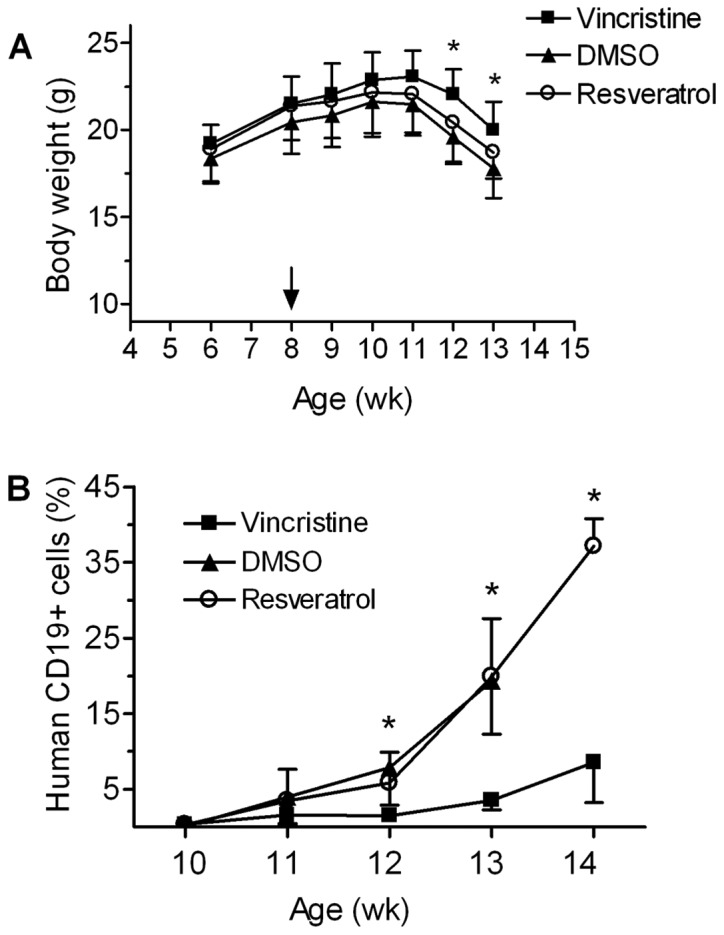
Loss of body weight was associated with increasing leukemia burden. Three groups of mice were engrafted with the t(4;11) ALL line SEM, and then treated intraperitoneally with either DMSO or resveratrol (10 mg/kg body weight) every other day, or vincristine (0.5 mg/kg body weight) three times per week for 4 weeks (n=16 per treatment group). (A) Body weight for each mouse was measured weekly beginning at age of 6 weeks. The asterisks indicate a difference in body weight for vincristine-treated mice compared to DMSO and resveratrol-treated mice (p<0.05). The arrow denotes the age of mice when the leukemia cells were injected. (B) PBLs were isolated from each mouse and stained with PE-Cy7 conjugated anti-human CD19 and APC-Cy7 conjugated anti-mouse CD45. The proportion of human CD19^+^ cells in the murine PBL population was monitored weekly by flow cytometry beginning 2 weeks after the injection of leukemia cells (age of 10 weeks) until the treatment period ended. The asterisks indicate a difference in the percent of CD19^+^ in the DMSO and resveratrol-treated mice compared to vincristine treated mice (p<0.05). Data represent means ± SD.

**Figure 4:**
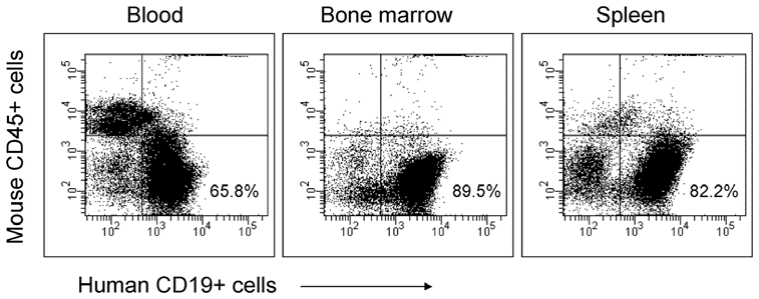
Engraftment sites for the SEM t(4;11) leukemia cells. Mice were engrafted with the t(4;11) ALL line SEM and treated with DMSO every other day for 4 weeks. Blood, spleens, and bone marrow were collected from 4 mice in the DMSO control group following euthanasia. Splenocytes were prepared by first perfusing with tissue with medium, and shredding to release the cells. The red blood cells were lysed in the blood and splenocyte preparations with PharmLyse. Both femurs from each mouse were removed into medium and the bone marrow was removed by perfusing the inside of the bone with medium. PBLs, splenocytes, and bone marrow cells were stained with PE-Cy7 conjugated anti-human CD19 and APC-Cy7 conjugated anti-mouse CD45 and analyzed by flow cytometry. Data are representative of four mice.

Other measured parameters showed the course of disease in these mice. Weekly body weight measurements showed no difference between the DMSO and resveratrol treated mice and loss of body weight began at ~3 weeks after injection of the leukemia cells in both groups (age 11 weeks, Fig. 3A). Vincristine-treated mice weighed more than the DMSO control mice at 12 and 13 weeks of age (20.0±1.6 vs 17.8±1.7 g, respectively at 13 weeks of age, p<0.05). The increasing burden of human CD19^+^ cells in peripheral blood of the mice corresponded to a reduction in body weight and the beginning of clinical illness (Fig. 3B). The percents of human CD19^+^ cells in the blood of DMSO and resveratrol treated mice were similar and were significantly higher than for vincristine treated mice from 12–14 weeks of age (p<0.05). Following euthanasia, engraftment sites of the SEM cells were evaluated in four mice from the DMSO control group. As expected for this model, substantial numbers of SEM cells were observed in bone marrow and spleen (>80% of total leukocytes), as well as the significant presence of the engrafted cells in the peripheral blood (Fig. 4).

### Resveratrol and metabolite levels in the sera

**Figure 5:**
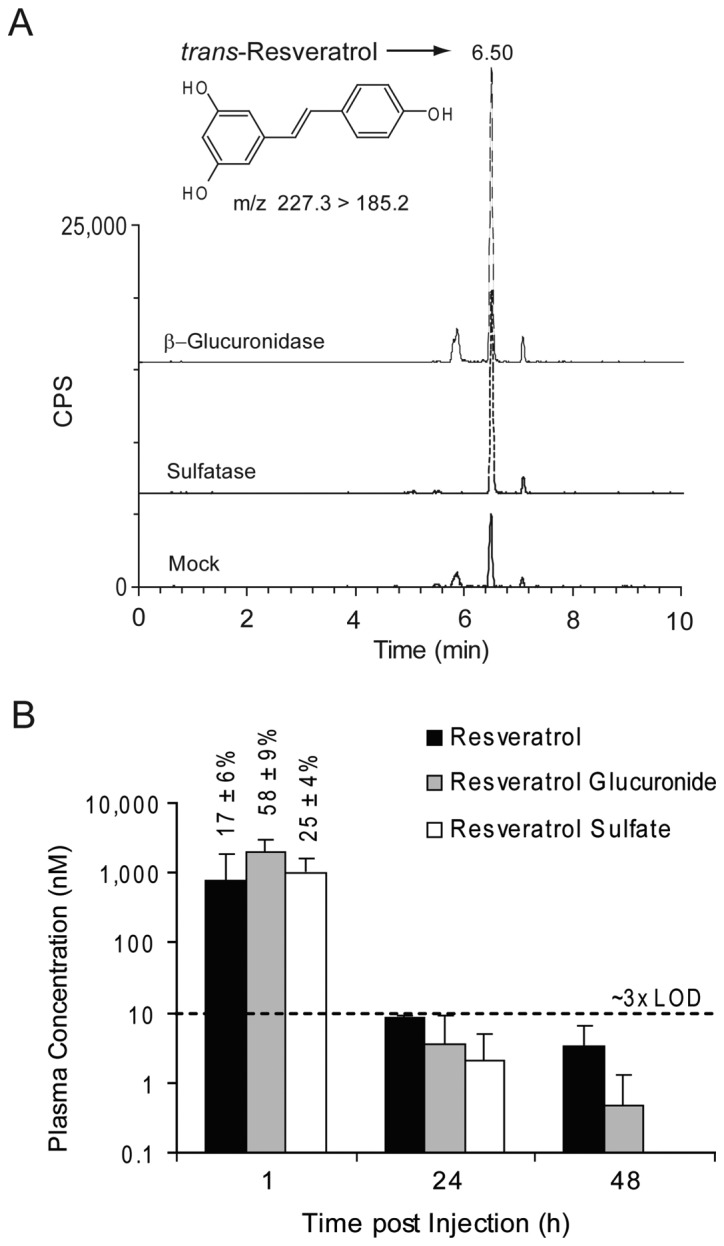
Leukemic NOD/SCID mice retain the ability to metabolize resveratrol. (A) Representative UPLC-MS/MS chromatograms of resveratrol isolated from serum collected 1 h post resveratrol i.p. injection after digestion with buffer (i.e., mock), sulfatase, or β-glucuronidase. Deconjugation reactions increased peak areas of resveratrol in serum relative to mock digestions demonstrating the rapid generation of glucuronide and sulfate conjugates *in vivo*. (B) At 1 h post-injection of resveratrol, mean plasma concentrations of total resveratrol metabolites were estimated at ~4±2 μM from 5 animals, with 17/58/25% distribution between parent, glucuronidated and sulfated metabolites. Data were presented as percent ± SEM. Concentrations were below the level of accurate quantification within 24 h, and the frequency of target analyte detection reduced from 67 to 30% between 24 and 48 h post-injection. LOD, limit of detection.

To evaluate resveratrol metabolism in the leukemic NOD/SCID mouse, surviving mice from the resveratrol group that were showing signs of clinical illness were injected i.p. with resveratrol, and euthanized at 1 h (n=5), 24 h (n=2), and 48 h (n=3) post injection. Results are summarized in Fig. 5. Assay performance was routinely acceptable. The 4-nitrophenyl conjugate controls indicated >99% deconjugation efficiency, while d4-resveratrol recoveries were 68±6, 68±6 and 86±10% for the mock, β-glucuronidase and sulfatase digestions, respectively.

As shown in Fig. 5A, sera incubation with either β-glucuronidase or sulfatase increased extractable resveratrol peak intensities. At 1 h post injection, serum concentrations of total resveratrol were ~4±2 μM, roughly distributed in a 1:3:1 ratio of resveratrol:resveratrol glucuronide:resveratrol sulfate (Fig. 5B). While traces of these metabolites were observed at 24 and 48 h, concentrations were <3x the instrumental detection limit of 3.7 nM, impacting their quantitative accuracy. Additionally, the frequency of compound detection decreased over time (1 h = 15/15 or 100%; 24 h = 4/6 or 67%; 48 h 3/9 or 30%). Thus, the leukemic NOD/SCID mouse definitively retained the ability to metabolize resveratrol, and at the 10 mg/kg dose, plasma concentrations of resveratrol were <10 nM within 24 h of i.p. injection.

## Discussion

In a previous study, we reported that resveratrol was efficient at killing t(4;11) ALL cells, as well as ALL cells without the translocation *in vitro* (22). However, in the current study resveratrol did not reduce or delay the growth of the t(4;11) ALL cells in NOD/SCID mice even when it was present at a concentration 20-fold greater than vincristine, a chemotherapeutic agent used in the clinical treatment of t(4;11) ALL (23). To our knowledge, this is the first report that i.p. administration of resveratrol does not kill t(4;11) ALL *in vivo*. The SEM cell line used for this study was established from a relapsed patient with ALL containing the translocation t(4;11) (11). We recently reported that this leukemia line is sensitive to vincristine *in vitro*, but becomes resistant to vincristine treatment *in vivo* due in part to the increased expression of the multi-drug resistant protein P-glycoprotein (25). In this same study, we found that vincristine was not toxic when administered to mice 3 times per week at a dose of 0.5 mg/kg body weight rather than once per week as is done for humans. In the present study, vincristine was able to impede the growth of the SEM leukemia cells for 7–14 days compared to the control treatment. Therefore, even though multi-drug resistant proteins may interfere with efficacy of therapeutic agents against this cell line over time, resveratrol was expected to show some ability to inhibit leukemia cell growth in the NOD/SCID mouse model, especially at a dose that was considerably higher than vincristine.

Other investigators using different cancer models have reported efficacy of resveratrol in inhibiting tumor cell growth. In a study of ovarian cancer using Balb/c nu/nu mice, resveratrol administered by daily i.p. injection at concentrations of 50 and 100 mg/kg body weight for 4 weeks reduced the growth of a subcutaneously xenografted ovarian tumor (26). At a concentration of 20 mg/kg body weight, daily i.p. administration of resveratrol was reported to inhibit the growth of subcutaneously xenografted bladder cancer (27). In the subcutaneous growth of tumors from Erlich’s ascites, resveratrol at 20 and 40 mg/kg body weight was able to inhibit growth of the tumor after 20 consecutive days of i.p. injections in mice (28). Neuroblastoma tumor growth, also subcutaneous, was similarly reported to be reduced when 5 mg resveratrol (per injection) was injected around the tumor or directly into the tumor (29).

In the present study, the dose of resveratrol in the NOD.CB17-Prkdc^scid^/J mice was limited to 10 mg/kg body weight due to unexpected toxicity observed at 20 and 40 mg/kg body weight which resulted in high mortality. It is unclear why the leukemic mice did not tolerate the higher doses of resveratrol. This intolerance of leukemic NOD/SCID mice to concentrations of resveratrol used in other cancer prevention studies may be reflective of the engraftment sites and systemic illness induced by this disease compared to subcutaneous xenograft models, and/or the intolerance may be mouse strain-dependent. Sites of infiltration of childhood ALL include liver, spleen, kidney, and central nervous system (30). Oral resveratrol has shown renal toxicity in rats at a dose of 3 g/kg bodyweight (31), so it is possible that mice with leukemic burden in non-hematological organs, such as liver or kidney, may have an increased sensitivity to resveratrol toxicity after i.p. injection.

Studies have been performed in both humans and animals to determine the tissue distribution, excretion rates, and the general bioavailability of resveratrol. The majority of bioavailability studies have been performed after oral administration. For example, ^14^C-labelled resveratrol at a single oral dose of 5 mg/kg body weight was detected in the duodenum, colon, liver, kidney, lung, spleen, heart, brain, and testis of mice by 3 h (32). Intraperitoneal injection of resveratrol at a dose of 20 mg/kg body weight resulted in the predominant presence of both sulfate and glucuronide conjugates in mouse serum (33). In these experiments, 13 μM resveratrol sulfate and 5 μM resveratrol glucuronide were detected in the serum 15 min after i.p. injection, with concentrations decreasing over the next 2 h. In another study with gerbils, i.p. injection of resveratrol at a concentration of 30 mg/kg body weight resulted in approximately 20 μM resveratrol in the serum after 1 h with a decline to less than 1.5 μM after 24 h, with little detectable resveratrol present after 48 h (34). The authors reported the resveratrol was present mainly as the glucuronide form. Our data show a similar pattern of presentation of resveratrol in the serum of leukemic mice, in that the resveratrol was rapidly metabolized to glucuronidated and sulfated conjugates within the first hour after i.p. injection, with a 1:3:1 distribution in the aglycone/gluronide/sulfate forms at that time. By 24 h, serum concentrations of both resveratrol and its metabolites were less than 10 nM.

The data presented in this study and the bioavailability studies described above show the importance of the metabolic products and the biological clearance of these agents when assessing the potential of phytochemicals as chemopreventive agents against leukemia. Recent reports on the biological activity of resveratrol metabolites showed that resveratrol sulfates can inhibit nitric oxide production, demonstrate radical scavenging activity, and inhibit cyclooxygenase-1 and -2 activities, whereas glucuronides had potent antioxidant potential *in vitro* (35–37). Resveratrol sulfates were also cytotoxic to breast cancer cells *in vitro*, but they were approximately 3–4-fold less toxic than resveratrol (38). However, the cytotoxicity observed in the latter study was at non-physiologic concentrations with an IC_50_ concentration of resveratrol at more than 60 μM, questioning the usefulness of these types of experiments. Parenterally administered resveratrol is metabolized to conjugated chemical forms that are either not cytotoxic or not at sufficient concentrations to induce apoptotic cell death in t(4;11) ALL cells *in vivo*. Caution should be exercised in future *in vitro* and *in vivo* research with phytochemicals for the potential prevention of t(4;11) or other ALLs, and researchers will need to consider the effects of metabolic alterations that occur *in vivo*. Finally, we note that while resveratrol by itself was not efficacious in the current study, it has been argued that resveratrol may be of value as a complementary agent in the treatment of select cancers (39). This is a possibility that clearly merits additional study with respect to the potential value of resveratrol in treating ALL. However, it is equally important to note that resveratrol has been reported to blunt the actions of select chemotherapeutic agents, such as proteasome inhibitors and paclitaxel (40,41). The above underscores the need to not only consider the impact of absorption, distribution, metabolism, and excretion of resveratrol and its subsequent biological actions, but also the possibility of resveratrol having deleterious side effects.

## Figures and Tables

**Table I:** Analyte specific API 4000 QTrap MS/MS^a^ and control sample parameters.

Analyte	Precursor (m/z)	Product (m/z)	+DCP (V)	+CE (V)	Spiked normal serum (μM)
Resveratrol	227.32	185.21	−80	−30	0.60
Resveratrol-d4	231.32	188.21	−80	−30	2.0
Resveratrol-3-O-D-glucuronide	403.32	227.32	−15	−37	0.20
Resveratrol-4′-O-D-glucuronide	403.32	227.32	−15	−37	0.20
Resveratrol-3-O-sulfate	307.32	227.32	−40	−30	0.20
Potassium4-nitrophenyl sulfate	218.2	138.2	−50	−30	0.50
4-Nitrophenyl β-D-glucuronide	314.2	138.2	−50	−30	0.50
CUDA	339.36	214.2	−65	−30	-

a 4000 QTrap parameters: ionspray voltage, 4.5 kV; curtain gas flow, 40 ml/min; collision gas, high; heater and source temperatures, 500°C; EP/CXP - 10 V; Gas 1/Gas 2, 40 ml/min.
